# Narrative Review of Single-Port Surgery in Genitourinary Cancers

**DOI:** 10.3390/cancers17030334

**Published:** 2025-01-21

**Authors:** Olamide Omidele, Yuval Elkun, Christopher Connors, Ahmed Eraky, Reza Mehrazin

**Affiliations:** Department of Urology, Icahn School of Medicine at Mount Sinai, New York, NY 10029, USA; yuval.elkun@mountsinai.org (Y.E.); christopher.connors@icahn.mssm.edu (C.C.); ahmed.eraky@mountsinai.org (A.E.); reza.mehrazin@mountsinai.org (R.M.)

**Keywords:** single-port, urologic oncology, radical prostatectomy, radical cystectomy, nephrectomy, transvesical, retroperitoneal, extraperitoneal

## Abstract

Background: The da Vinci single-port (SP) platform is emerging as the latest innovation in minimally invasive surgery and its utilization in treating urologic malignancies continues to expand. Methods: A search was conducted in PubMed, MEDLINE, and ScienceDirect. The final set includes 40 academic articles. Results: Research on single-port surgery for genitourinary cancer is still an emerging topic. We divided the topic into the following categories: radical prostatectomy, radical cystectomy, nephrectomy, and nephroureterectomy. Conclusions: The single-port platform provides urologists with another tool to tackle more complex surgical cases and pathologies with the added improvements of decreased length of stay and increased pain tolerance for patients.

## Simple Summary

The current paper highlights the current literature on single-port surgery for genitourinary cancers. The authors aim to achieve a comprehensive review of the topic that can serve as a guide to urologic surgeons interested in learning about the advantages and disadvantages of this novel technology. Single-port technology allows for a more diverse approach to complex urologic surgery with the additional benefit of improved recovery due to decreased postoperative pain. As the field continues to advance, reviews such as this will be important catalysts for further surgical innovations that will enhance patient outcomes.

## 1. Introduction

An estimated 20% of all new cancer diagnoses in the United States derive from urologic malignancies [1]. Reports from the Global Burden of Disease Study show a 2.1-fold increase in kidney cancer, a 1.5-fold increase in bladder cancer, and a 3.2-fold increase in prostate cancer projected over the next few decades [2]. As the prevalence of these cancers continues to increase, we continue to find better ways to provide surgical care to these patients to not only provide adequate oncologic control, but to also minimize adverse effects. Multi-port (MP) da Vinci Xi and Si (Intuitive Surgical, Sunnyvale, CA, USA) are now the most widely used robotic systems in urologic procedures [3,4]. Many reports have shown improved perioperative outcomes, including decreased surgical morbidity and convalescence time for patients undergoing minimally invasive surgical approaches, compared to the standard open approaches [5,6]. These reports have been validated in the urologic oncology literature for the past decade. The single-port (SP) da Vinci system was approved by the United States Food and Drug Administration (FDA) in 2018, thus further minimizing the impacts of minimally invasive surgery. The advantages of the SP platform are numerous but largely hinge on the fact that it requires only a single incision, which helps to reduce postoperative pain and wound complications. Additionally, the flexibility of the camera and instruments, utilizing a “two points of articulation” mechanism, allows the surgeon to operate in a smaller surgical field. This report highlights SP utilization and outcomes for urologic oncologic procedures in the hope of providing insight into early surgeon experience with the technology in the treatment of prostate, bladder, and kidney cancer.

## 2. Radical Prostatectomy

By 2013, 85% of prostatectomies in the United States were performed robotically, with controversial reports supporting improved erectile performance postoperatively as well as lower rates of urinary incontinence when compared to other approaches [7,8]. Many documented approaches to single-port robotic-assisted radical prostatectomy (SP-RARP) have been reported, including transperitoneal, extraperitoneal, Retzius sparing, and transvesical approaches (Table 1). Kaouk et al. were the first to publish their findings on a series of 11 patients who underwent SP-RARP in 2014, reporting low operative times, minimal blood loss, and no intraoperative complications with the SP platform [9]. This review will focus on transperitoneal, extraperitoneal, and transvesical approaches to RARP.

### 2.1. Transperitoneal SP-RARP

There are many techniques described for transperitoneal SP-RARP trocar placement, but most elect for placing the trocar in the midline above the umbilicus, roughly 20 cm away from the pubic bone. Some surgeons also place a 12 mm assistant trocar in the right lower quadrant [11]. Covas Moschovas and colleagues described the difference between the SP and MP platforms for radical prostatectomy. They noted that the SP platform needs a greater working distance, with constant use of the relocation pedal to target the robot toward the operative field. Furthermore, the SP platform has lower strength compared to the MP platform, which results in lower traction and tissue-grasping ability.

Noh et al. published a series comparing SP-RARP vs. multiport RARP (MP-RARP) [12]. They retrospectively reviewed 40 consecutive SP-RARP patients compared to 129 MP-RARP patients. Noh et al. conducted propensity score matching, noting no significant difference in perioperative parameters such as operative time, estimated blood loss (EBL), and positive surgical margins. However, SP-RARP was associated with a lower proportion of complete nerve sparing as compared to MP RARP, which Noh et al. attributed to the inherent limitations of SP technology, including its reduced tissue grasping and traction ability. Another study by Abaza et al. looked at the early experience of two centers with SP-RARP systems [10]. Similar to the rest of the literature, they found steady improvements in operative time and EBL. Interestingly, Abaza et al. noted an increased rate of same-day discharge in one of the operative groups, with 88% of patients discharged on the same day of surgery. The high rate of same-day discharge was attributed to faster recovery, less postoperative pain, and short hospitalization, associated with a single incision.

### 2.2. Extraperitoneal

The merits of the extraperitoneal approach are best observed in patients with previous abdominal or pelvic surgery, where one may encounter a significant amount of adhesions. Additional advantages with the extraperitoneal approach include minimal peritoneal irritation and bowel manipulation, less steep Trendelenburg, and less pneumoperitoneum [14]. These factors directly impact postoperative gastrointestinal symptoms such as nausea and vomiting but also indirectly impact the length of stay in the hospital due to improved pain parameters. The extraperitoneal approach is ideal for obese patients, who are poor candidates for the steep Trendelenburg position, as well as those with previous abdominal surgeries [15]. Patients who may not be ideal candidates for an extraperitoneal approach include those with prior inguinal surgery, such as those with prior hernia repairs with mesh or kidney transplant.

Zeinab et al. evaluated the perioperative and early postoperative outcomes for transperitoneal vs. extraperitoneal approaches in SP-RARP [13]. The extraperitoneal group was found to have significantly higher operative times (206 min vs. 155 min; *p* < 0.001). The greater operative time in the extraperitoneal group was attributed to differences in surgeon comfort as well as greater rates of lymph node dissection performed in the extraperitoneal group (84.6% vs. 52.9%, *p* < 0.001). Additionally, the extraperitoneal group was noted to have a shorter median length of hospital stay (7 h), resulting in a 53% same-day discharge rate as compared to 1% in the transperitoneal group. The transperitoneal group had greater rates of postoperative gastrointestinal side effects, contributing to a longer median length of hospital stay. Functional outcomes such as continence rates were comparable between the two groups at 6 weeks, 3 months, and 6 months. Lymph node yield was higher in the transperitoneal group but did not reach statistical significance (*p* = 0.066).

### 2.3. Transvesical

One of the main advantages of the SP platform is the ability to operate in small anatomical spaces, making a transvesical approach possible. Zhou et al. previously described the surgical technique for transvesical MP-RARP, with a 95% early continence rate reported [16]. The positive surgical margin rate was comparable to what has been previously reported in the literature, at 11%. However, the technique is slightly different, involving a posterior bladder incision to enter the intravesical space. There are limitations associated with transvesical SP-RARP, including difficulty with extended pelvic lymph node dissection and large prostate size.

Kaouk et al. were the first to describe the transvesical SP-RARP technique, reporting a 95% continence rate within 2–7 days post catheter removal [17]. The positive surgical margin rate was 15%, consistent with most published case series. As the study assessed only 20 patients, the authors noted that the low sample size was a limitation, and further studies are needed to characterize the benefits of transvesical SP-RARP.

## 3. Radical Cystectomy

Historically, open radical cystectomy (ORC) with pelvic lymph node dissection has been the gold standard treatment for high-risk, non-muscle invasive, and localized muscle-invasive bladder cancer [18,19]. Robotic-assisted RC (RARC) was first described in 2003 but has only recently gained popularity following technique standardization and several landmark studies comparing ORC and RARC [18,20]. The RAZOR trial, published in 2018, was a prospective noninferiority trial demonstrating comparable oncologic outcomes between patients undergoing MP-RARC and ORC [21]. Similar to other published series in the literature, minimally invasive RC resulted in lower rates of perioperative blood transfusions and reduced hospital stay [21,22,23].

The SP platform carries significant advantages such as camera flexibility, fewer incisions, and improved robot patient-cart maneuverability for multi-quadrant surgery (e.g., pelvic for cystectomy and right lower quadrant for bowel harvest) [24]. One of the major initial concerns with performing SP-RARC was in relation to intracorporal or extracorporeal diversion. The technique of SP-RARC with intracorporeal ileal conduit urinary diversion performed at our institution is similar to that described by Kaouk et al. in 2019 [25]. Male patients are placed in the supine position, while female patients are placed in the dorsal lithotomy position to allow for easier access to the vagina while maintaining that all pressure points are padded. Port placement is similar to that of SP-RARP, except for an additional assistant port in the right lower quadrant for ileal conduit formation, where the final stoma will be created. SP-RARC techniques with extracorporeal diversions have also been described. Fang et al. performed extracorporeal diversions in 47 SP-RARC patients, in which they found that the extraction of the specimen required them to open the initial SP port incision, which conveniently allowed them to perform the urinary diversion extracorporeally [26]. For patients who underwent an ileal conduit, the surgeon’s technique involved utilizing the assistant port to bring the ileal conduit through, resulting in only one incision closure required in their patients.

Kaouk et al. were one of the first groups to report on SP-RARC in 2019, evaluating outcomes in a case series of four patients who underwent SP-RARC with intracorporeal urinary diversion. No Clavien–Dindo (CD) grade II or greater complications within 30 days postoperatively were reported [25]. Shortly after, Zhang et al. also published a series of four patients who underwent successful SP-RARC, which further affirmed the feasibility of this approach [27]. Given that prior studies on MP-RARC have noted postoperative CDII complication rates as high as 50%, both of these initial studies suggested that SP-RARC may not increase the risk of complications, even during the initial surgical learning curve [21,28].

In 2021, Gross et al. published a 1:2 propensity-matched analysis of 12 patients undergoing SP-RARC with intracorporeal diversion compared to those treated with MP-RARC. They found that both platforms resulted in similar estimated blood loss, operative times, 90-day complication and readmission rates, and rates of positive surgical margins on final pathology [29]. However, they did note a significantly lower lymph note yield in the SP-RARC group (11.9 nodes vs. 17.1 nodes for MP-RARC patients). Subsequently, Ali et al. published a study in 2022 comparing 14 patients undergoing SP-RARC with intracorporeal diversion against 20 patients undergoing MP-RARC [30]. They found no significant differences in rates of complications, readmissions, or positive surgical margins between the groups. Notably, similar to Gross et al., they also found a lower mean lymph node yield in the SP-RARC group (16 vs. 22 nodes), although this difference was not significant. In line with other published reports on the SP approach, Ali et al. found that SP patients had significantly less postoperative narcotic use (11.5 vs. 25 morphine milligram equivalents) and a significantly quicker return of bowel function after surgery (2 vs. 3 days) [30].

More recently, Fang et al. published an analysis of 47 patients who underwent SP-RARC with an extracorporeal urinary diversion compared to 49 MP-RARC patients who underwent either an intracorporeal or extracorporeal urinary diversion [26]. In the largest study evaluating SP-RARC thus far, Fang et al. found no difference in the rates of postoperative complications, readmissions, disease recurrence, or total length of hospitalization between groups. Of note, while postoperative narcotic use was similar between groups, SP-RARC patients had a significantly faster return of bowel function compared to MP-RARC patients (3.4 vs. 4.5 days, *p* < 0.01).

Altogether, these early studies suggest that SP-RARC is a feasible alternative to MP-RARC, as evidenced by similar complication rates, readmission rates, EBL, operative times, and length of hospitalization (Table 2). Furthermore, the SP platform may confer additional benefits over MP-RARC, such as decreased narcotic use and faster return of bowel function. However, SP-RARC has been consistently shown to be associated with a lower lymph node yield, warranting further investigation as lymph node yield is a useful predictor of progression-free and overall survival [31,32]. There are no society recommendations favoring SP-RARC over MP-RARC, as the availability of the SP robot remains limited worldwide. Future large multicenter trials are needed to evaluate the oncologic benefits, functional outcomes, and cost efficiency of SP-RARC relative to MP-RARC. In the interim, SP-RARC can be considered in patients with a hostile abdomen and limited working space where an open approach may prove to be technically challenging.

## 4. Nephrectomy

Robotic-assisted nephrectomy, whether performed as a partial nephrectomy (RAPN) or radical nephrectomy (RARN), has risen to the forefront as the standard of care for the surgical management of renal tumors. Compared to open or laparoscopic nephrectomy, robotic-assisted nephrectomy is associated with shorter hospital length of stay, lower EBL, shorter warm ischemia time, and improved early renal function preservation [33].

Currently, there are no society guidelines regarding patient or tumor selection for MP- versus SP-RAPN or RARN. Recently, Razdan et al. attempted to provide clarity on this topic, proposing an algorithm for selecting a surgical technique based on tumor characteristics [34]. In the proposed algorithm, Razdan et al. define tumor complexity based on the R.E.N.A.L score in combination with the amount of visceral or retroperitoneal fat present to determine whether patients should have an SP or MP procedure. Of note, all patients considered for RARN, with high tumor complexity based on the R.E.N.A.L nephrometry score or with high volumes of visceral or retroperitoneal fat, were recommended to undergo an MP surgical approach. Patients with low or intermediate-complexity anterior tumors, a low volume of visceral or retroperitoneal fat, and a prior history of significant abdominal surgery could undergo SP surgery using either transperitoneal or retroperitoneal approaches. If patients with low or intermediate-complexity tumors and low volumes of fat had posteriorly located tumors or a history of prior abdominal surgery, an SP retroperitoneal approach was preferred [34].

### 4.1. Partial and Radical Nephrectomy

The da Vinci SP robotic system has been shown in numerous studies to be non-inferior to the da Vinci MP robotic system when performing RAPN (Table 3). Okhawere et al. reported no significant differences in EBL, operative time, the positive surgical margin rate, and complication rates between SP- and MP-RAPN [35]. However, there was a statistically significant difference in the longer mean ischemia time with the single-port cohort. In a recent meta-analysis comparing outcomes between SP- and MP-RAPN, Nguyen et al. found no significant differences between surgical platforms in terms of intra- and postoperative complications, the pain score and morphine milligram equivalent usage, hospital stay, positive surgical margin rates, and postoperative eGFR [36]. Additionally, the single-port group was associated with a significantly longer ischemia time, less EBL, higher blood transfusion rate, and higher postoperative eGFR at 6 months postoperatively.

While a majority of nephrectomies performed using the da Vinci SP platform are partial nephrectomies, SP-RARN has been described using the same operative techniques as with partial nephrectomy. Fang et al. describe their initial clinical experience with SP-RARN and SP-RAPN, with no adjustment in surgical technique between the two [37]. Fang et al. utilized an Endo-GIA stapler to staple across the renal vein and artery prior to the excision of the kidney. Of the 16 patients in their study, 3 underwent a radical nephrectomy. Fang et al. reported no significant differences in tumor size, operative time, EBL, or immediate postoperative complications between the partial and radical nephrectomy groups.

### 4.2. Retroperitoneal and Low Anterior Access (LAA)

As experience with the da Vinci SP platform has grown since its introduction in 2018, surgeons have begun to develop alternative access methods for the SP robotic system. Recently, several groups have introduced a low anterior access (LAA) incision for SP-RAPN, enabling either a transperitoneal or retroperitoneal approach to be performed through the same incision [39,40]. LAA incision was coined by the Single Port Advanced Research Consortium but has previously been referred to in the literature as Single Port Ahmed Modification (SPAM), supine anterior retroperitoneal access (SARA), and lower anterior retroperitoneal access (LARA). With the LAA incision technique, the patient is positioned in a lateral decubitus position. The distance between the ipsilateral anterior superior iliac spine (ASIS) and the umbilicus is marked, and a 3 cm incision is made at 1/3 of the distance between the two landmarks, closer to the ASIS (also known as McBurney’s point). Dissection is then performed until the transversalis fascia is reached. At this point, the surgeon decides whether to proceed with a transperitoneal or retroperitoneal approach to the partial nephrectomy. If a transperitoneal approach is chosen, the peritoneum is opened, the access port is inserted, the pneumoperitoneum is achieved, and the procedure is performed as described above. If a retroperitoneal approach is chosen, the peritoneum is not violated, and blunt dissection is used to open the retroperitoneal space before introducing the access port. Crivellaro et al. utilized the LAA retroperitoneal technique, which they referred to as the SARA technique, to perform 14 nephrectomies (12 partial, 2 radical), with no reported differences in intra-operative or postoperative outcomes between the two groups [39].

## 5. Nephroureterectomy

Nephroureterectomy with ipsilateral ureteral orifice and bladder cuff excision has long been the standard of care for most cases of upper tract urothelial carcinoma (UTUC) [41,42]. As comfort with the da Vinci SP robotic system has grown, SP robotic nephroureterectomy (RANU) utilizing both transperitoneal and retroperitoneal techniques, has been described in the literature. However, there is still a paucity in the literature of descriptions of outcomes with SP-RANU.

Several limitations with RANU utilizing the MP robotic system have paved the way for the adoption of the SP robotic system for this procedure. Nephroureterectomy is a unique surgery for urologists as it is one of a handful of procedures performed in multiple abdominal quadrants. Historically, this often necessitated intra-operative patient repositioning and redocking of the robot following the radical nephrectomy portion of the procedure to isolate the distal ureter and perform the bladder cuff excision. However, the transition from the da Vinci Si robotic system to the Xi robotic system has significantly reduced the incidence of repositioning and redocking [43]. Furthermore, as surgeons have begun to transition to retroperitoneal approaches to kidney surgery due to lower EBL, shorter hospital stays, and the ability to avoid the intra-abdominal cavity in patients with significant past surgical history compared to transperitoneal approaches, the MP robotic system faces new challenges. The retroperitoneum offers limited working space, which can result in external instrument clashing and limited instrument maneuverability, which may be improved when using the SP robotic platform.

Pellegrino et al. describe their initial experience performing SP-RANU utilizing both the transperitoneal and retroperitoneal approaches [42]. As this is still a relatively new procedure, they recommend further studies to characterize the oncologic benefit of this approach, citing technical challenges associated with lymph node dissection. Bang et al. began performing retroperitoneal SP-RANU in 2021 and published a series of twenty patients [41]. The median operating time was 150 min, the EBL was 122 mL, and no intraoperative complications were reported. The authors of this report recognize limitations surrounding generalizability in relation to outcomes published in their study, as the surgeries were performed by a single surgeon with significant experience with SP approaches. Nonetheless, early reports support SP-RANU as a viable approach to UTUC in the right patient (Table 3).

## 6. Conclusions

As the prevalence of urologic cancers continues to increase, urologists must continue to develop new surgical approaches and techniques to keep up with an ever-changing field. Though not devoid of shortcomings, the SP robotic platform provides urologists with another tool in their armamentarium to tackle more complex surgical cases and pathologies. Further studies are required to fully understand the true benefit of this technology as it relates to urologic cancers, but we hope this review provides a sounding board on the potential of SP robotic systems to treat a variety of urologic cancers.

## Figures and Tables

**Table 1:** The benchmark single-port prostatectomy literature.

Surgery Type	Author	Year	Journal	Description/Objective	Study Design	# Patients
Prostatectomy	Kaouk et al. [9]	2014	European Urology	The first clinical investigation of single-port robotic surgery for urologic procedures	Single-institution prospective case series	19 (11 RARP)
	Abeza et al. [10]	2020	Journal of Endourology	A description and comparison of outcomes between two distinct methods for adopting SP-RARP by two experienced surgeons	Two-institution prospective cohort study	74 (34 and 40)
	Moschovas et al. [11]	2022	Journal of Endourology	Assesses the outcomes and factors influencing the initial learning curve of single-port robot-assisted radical prostatectomy	Single-institution retrospective cohort study	100
	Noh et al. [12]	2022	Journal of Endourology	A comparison of a series of MP-RARPs and SP-RARPs performed by a single surgeon	Single-institution retrospective cohort study	40 SP-RARP, 129 MP-RARP
	Zeinab et al. [13]	2023	Urology	A comparison of outcomes between extraperitoneal and transperitoneal SP-RARP	Multi-institution retrospective cohort study	476 (238 per arm post-matching)

**Table 2:** The benchmark single-port cystectomy literature.

Surgery Type	Author	Year	Journal	Description/Objective	Study Design	# Patients
Cystectomy	Kaouk et al. [25]	2019	BJU International	An initial description and evaluation of outcomes of a single-port technique for single-port RARC with intracorporal diversion and PLND	Single-institution prospective case series	4
	Zhang et al. [27]	2020	Translational Andrology and Urology	An initial description and evaluation of outcomes of a single-port technique for single-port RARC with intracorporal diversion	Single-institution prospective case series	4
	Gross et al. [29]	2021	Journal of Endourology	Aomparison of outcomes and analgesic requirements between SP- and MP-RARC with urinary diversion	Single-institution retrospective cohort study	96 (49 MP, 47 SP)
	Ali et al. [30]	2022	Journal of Endourology	An evaluation of perioperative outcomes between SP- and MP-RARC with intracorporal diversion	Single-institution prospective cohort study	34 (20 MP, 14 SP post-matching)
	Fang et al. [26]	2024	Journal of Endourology	An evaluation of perioperative outcomes between SP- and MP-RARC with intracorporal diversion	Single-institution retrospective cohort study	36 (24 MP, 12 SP post-matching)

**Table 3:** The benchmark single-port nephrectomy literature.

Surgery Type	Author	Year	Journal	Description/Objective	Study Design	# Patients
Radical and Partial Nephrectomy	Fang et al. [37]	2020	Journal of Robotic Surgery	An evaluation of the initial experience, techniques, and perioperative outcomes of SP-RAPN and SP RARN	Single-institution retrospective case series	16 (13 PN, 3 RN)
	Glaser et al. [38]	2022	Journal of Robotic Surgery	A comparison of outcomes and analgesic requirements between SP- and MP-RAPN	Single-institution retrospective cohort study	78 (52 MP, 26 SP)
	Okhawere et al. [35]	2022	Journal of Endourology	A comparison of perioperative outcomes between SP- and MP-RAPN	Multi-institution prospective cohort study	1726 (1578 MP, 148 SP)
	Pellegrino et al. [39]	2023	European Urology	An evaluation of a novel supine anterior retroperitoneal access technique for SP surgery including PN, RN, RNU, and pyeloplasty	Single-institution prospective cohort study	18 (12 PN, 2 RN, 2 RNU, 2 pyeloplasty)
	Rich et al. [33]	2023	European Urology Focus	A comparison of transperitoneal vs. retroperitoneal SP-RAPN	Multi-institution prospective cohort study	219
	Nguyen et al. [36]	2024	Journal of Endourology	An evaluation of perioperative, oncological, and functional outcomes between SP- and MP-RAPN	Meta-analysis	n/a
	Billah et al. [40]	2024	European Urology Focus	A description and evaluation of a novel lower anterior access technique for SP-RAPN	Single-institution prospective cohort study	78
	Bang et al. [41]	2023	Journal of Clinical Medicine	Initial experience and evaluation of retroperitoneal single-port RANU	Single-institution retrospective case series	20
Radical Nephroureterectomy						
